# Evolution of Macromolecular Docking Techniques: The Case Study of Nickel and Iron Metabolism in Pathogenic Bacteria

**DOI:** 10.3390/molecules200814265

**Published:** 2015-08-05

**Authors:** Francesco Musiani, Stefano Ciurli

**Affiliations:** Laboratory of Bioinorganic Chemistry, Department of Pharmacy and Biotechnology, University of Bologna, Viale G. Fanin 40, Bologna I-40127, Italy

**Keywords:** macromolecular docking, urease, urease metallocenter biosynthesis, NikR, Fur, *Helicobacter pylori*

## Abstract

The interaction between macromolecules is a fundamental aspect of most biological processes. The computational techniques used to study protein-protein and protein-nucleic acid interactions have evolved in the last few years because of the development of new algorithms that allow the *a priori* incorporation, in the docking process, of experimentally derived information, together with the possibility of accounting for the flexibility of the interacting molecules. Here we review the results and the evolution of the techniques used to study the interaction between metallo-proteins and DNA operators, all involved in the nickel and iron metabolism of pathogenic bacteria, focusing in particular on *Helicobacter pylori* (*Hp*). In the first part of the article we discuss the methods used to calculate the structure of complexes of proteins involved in the activation of the nickel-dependent enzyme urease. In the second part of the article, we concentrate on two applications of protein-DNA docking conducted on the transcription factors *Hp*Fur (ferric uptake regulator) and *Hp*NikR (nickel regulator). In both cases we discuss the technical expedients used to take into account the conformational variability of the multi-domain proteins involved in the calculations.

## 1. Introduction

Living organisms rely on protein-protein and on protein-nucleic acid interactions to perform their functions [1,2,3,4]. Considering only protein-protein binary interactions, this number can go from *ca.* 10,000 in *Escherichia coli* [5], to *ca.* 18,000 in higher plants of the *Arabidopsis* genus [6], to 150,000–500,000 in human cells [7,8]. Despite their evident importance, the interactions between macromolecules are not fully understood at the structural level. Indeed, only a fraction of the putatively analyzable unique protein-protein interfaces are so far available from high-resolution X-ray crystallography and nuclear magnetic resonance (NMR) spectroscopy [9]. Structural information can also be obtained from low-resolution experimental techniques, such as cryo-electron microscopy or small-angle X-ray scattering which, however, do not provide enough molecular details of the interacting surfaces [10]. Experimental techniques can thus be complemented by computational docking methods aimed to model the quaternary structure of complexes formed by two or more interacting macromolecules. Protein-protein complexes are the most commonly-attempted targets of such modelling [10,11,12,13,14,15], followed by protein-nucleic acid complexes [16,17,18]. In the classical (or *ab initio*) docking methods, the calculation itself only produces plausible candidate structures. These candidates must then be ranked *a posteriori* using scoring functions or validation/filtering procedures that use experimental data to identify structures that are most likely to occur in nature. In recent years, integrative and information-driven algorithms changed the classical *ab initio* docking procedure by encoding *a priori* information derived from experimentally-identified or predicted protein interfaces to drive the docking process [10].

Here we review and discuss the evolution of the techniques used by us and by other groups to study the interactions between macromolecules in two macro-test cases: (i) the prediction of the complexes formed by the accessory proteins involved in the activation of the nickel-dependent enzyme urease; and (ii) the prediction of protein-DNA complexes involving two bacterial transcriptional factor, namely the ferric uptake regulator (Fur) and the nickel regulator NikR, involved in cellular iron and nickel metabolism.

## 2. Macromolecular Docking Overview

**Scheme 1:**
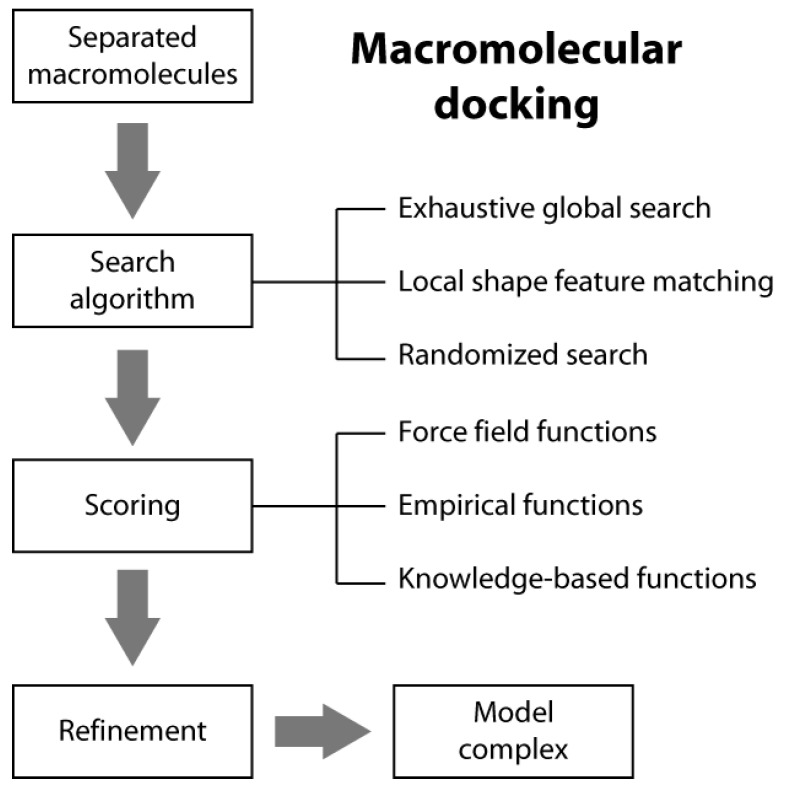
Macromolecular docking flow-chart.

Macromolecular docking is usually defined as the structural prediction of a molecular complex starting from the separated structures of its members. A typical docking algorithm involves three steps (see Scheme 1). The first step consists of the exploration of the large number of possible orientations and conformations that the members of a complex can assume in tridimensional space. This is usually done through a “search algorithm”. This exploration step is then followed by a scoring step of the resulting model complexes using an appropriate criterion. Finally, the procedure is often concluded by the refinement of a restricted number of the obtained structures of the complex.

### 2.1. Search Algorithms

In typical docking algorithms, one molecule is fixed (the so-called “static molecule”) and the other molecule is translated and rotated around (the so-called “moving molecule”). Depending on the search strategies, current *ab initio* docking programs can be divided into three general categories: (i) exhaustive global search; (ii) local shape feature matching; and (iii) randomized search algorithms [14].

The global search algorithms perform a scan over the three rotational and the three translational degrees of freedom. Given the huge number of possible orientations to be sampled, it is computationally prohibitive to evaluate all the binding complexes using a standard search method. To reduce the computational cost, two types of approaches have been developed for this type of exhaustive global search: fast Fourier transform (FFT) correlation and direct search algorithms [14]. The FFT-based algorithm accelerates the search process in the translational space by moving to the imaginary space through several FFT calculations. In the direct search algorithm approach, the molecular shape of two interacting molecules is first mapped onto a tridimensional grid and the shape matching is then directly performed in the Cartesian grid space to find the geometric fit between the two molecules.

In local shape feature-matching algorithms, the interacting molecules are represented by their shapes, such as the molecular surface. The search algorithms find those matches that give a good local shape complementarity between the two molecules. Local shape feature-matching algorithms also tend to generate more binding orientations towards those sites with good shape complementarity. Therefore, a clustering step is often necessary after the docking calculation to remove the redundancy in the final solutions [14].

In randomized search algorithms, the moving molecule is randomly placed around the binding site for a local search or around the whole static molecule for a global search based on a certain number of rules. The placement procedure can be improved by using additional information, such as the molecular surfaces, to generate more realistic initial binding orientations. Then, from their starting positions, each of the initially generated orientations is optimized and/or refined through a multistage sampling and/or multiscale modeling approach that use stochastic algorithms, such as genetic algorithms and/or Monte Carlo methods [14].

Docking search algorithms can use information extracted from experimental or bioinformatics sources to guide the formation of the complex. In this way, the sampling can be limited to the regions defined by the data and the number of unrealistic solutions can be dramatically reduced. On the other hand, the inclusion of experimental/prediction data does not guarantee that all complexes are correct [10].

### 2.2. Scoring Functions

There are three general classes of scoring functions: (i) force field; (ii) empirical; and (iii) knowledge-based, even if some algorithms use a combination of two or all of them [19].

In the case of force field scoring functions, the interaction affinities are estimated by considering physical atomic interactions, such as van der Waals and electrostatic interactions and bond stretching/bending/torsional forces [19]. Force field functions and parameters are usually derived from both experimental data and quantum mechanical calculations. Because the binding of the molecules normally takes place in the presence of water, the desolvation energies of the interacting molecules are sometimes taken into account using implicit solvation methods [19].

Empirical scoring functions estimate the binding affinity of a complex on the basis of a set of weighted energy terms [19]. The energy coefficients are determined by fitting the binding affinity data of a training set of protein-ligand complexes with known three-dimensional structures. Compared to the force field scoring functions, the empirical scoring functions are much faster in binding score calculations due to their simple energy terms.

The knowledge-based (also known as statistical potentials) scoring functions are based on statistical observations of intermolecular close contacts in structural databases that are used to derive “potentials of mean force”. This method is based on the assumption that close intermolecular interactions between certain types of atoms or functional groups that occur more frequently than one would expect by a random distribution are likely to be energetically favorable and therefore contribute favorably to binding affinity [20].

### 2.3. A Peculiar Case: Protein-DNA Docking

DNA is considered a difficult target for macromolecular docking because of its inherent flexibility summed with the scarcity of the information to define the DNA-binding interfaces. DNA can exhibit large conformational changes upon binding to a protein, which can greatly modify the shape of the interaction surface. As a direct consequence, the total conformational space that needs to be searched in order to find favorable conformations becomes too computationally expensive [21]. In the present review we discuss protein-DNA calculations performed using the program Haddock [22,23] and a two-stage docking approach specifically developed for the calculation of protein-DNA complexes [21]. These calculations yielded good performances [24] and are described in the examples discussed here. Haddock (High Ambiguity Driven biomolecular DOCKing) implements an approach that uses biochemical and/or biophysical interaction data to drive the docking process. The calculation is guided by defining selected residues as “active” in the protein-protein or protein-DNA interaction. The docking algorithm rewards the complexes that have these active residues on the interaction interface. Haddock simulations are composed of three rounds: (i) a rigid body energy minimization that produces a user-defined number of putative docking complexes (usually 1000); (ii) a semi-flexible simulated annealing carried out on the best solutions calculated in the first round and found on the basis of the intermolecular energy (habitually 200); and (iii) an explicit water refinement carried out on the same structures of the previous step. Haddock has been implemented by using the program CNS [25] for structure calculations and python scripts derived from ARIA [26] for automation. The solutions are then clustered on the basis of the pairwise backbone RMSD and further analyzed for structural and functional congruence. The cut-off for the clustering of protein-protein docking solution is typically set to 7.5 Å. The protein-DNA docking protocol developed for Haddock starts with the generation of a model for the unbound DNA operator using the DNA analysis and rebuilding software 3DNA implemented in the 3D-DART server [27]. In the first docking round, additional restraints are introduced for the DNA fragment to maintain base planarity and Watson-Crick bonds. Subsequently, the DNA conformation in the docked resulting structures are analyzed using the program DART [27] in order to determine trends in DNA bending and twisting, a type of information that is used to generate an ensemble of custom DNA models representing the accessible conformations. A second, and final, docking round is then carried out following the same approach described for the first round, but this time including the ensemble of pre-bent DNA models generated above and representing various degrees of conformational flexibility of the nucleic acid. In this round, the conformational freedom of the DNA molecule is restricted at the semi-flexible refinement stage (see below) in order to prevent helical deformation.

Here below we describe and discuss specific examples of macromolecular docking applied to proteins involved in the cellular metabolism of nickel and iron.

## 3. Urease Activation

**Figure 1:**
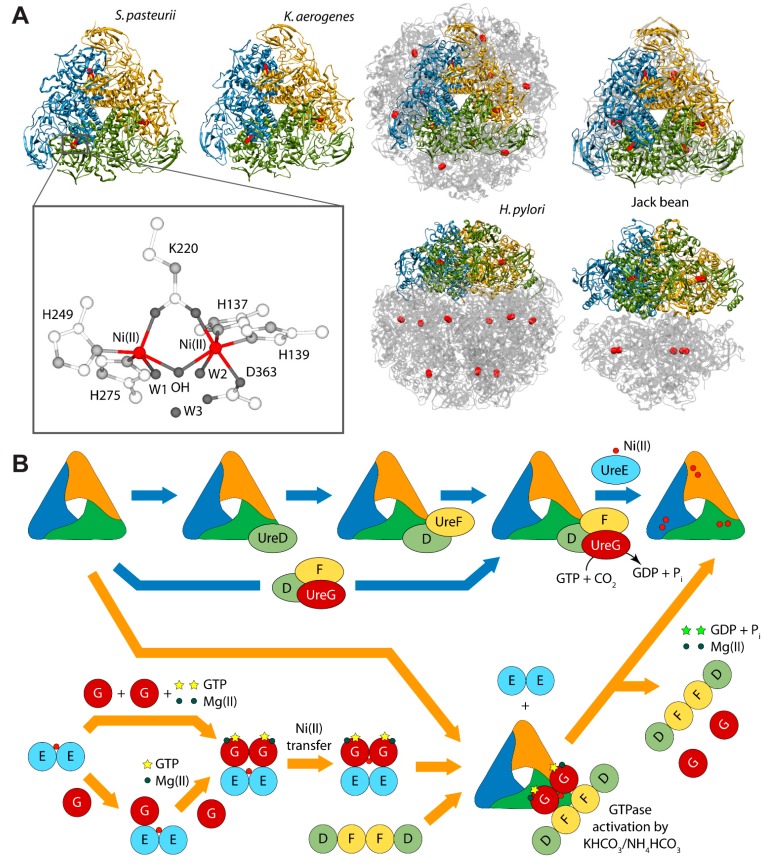
(**A**) Ribbon diagram of urease from *S. pasteurii*, *K. aerogenes*, *H. pylori*, and jack bean. Ribbons are colored to evidence the chains composing the protomers. Ni(II) ions are reported as red spheres. The proteins are seen through the ternary axis (top panels). The bottom panels are rotated by 90° around the horizontal axis *vs.* the top panels. The inset reports the details of Ni(II) coordination geometry of in native urease active site; (**B**) Schematic representation of the proposed mechanisms for urease activation.

Urease is a nickel dependent enzyme that catalyzes urea hydrolysis in the last step of organic nitrogen mineralization to give ammonia and carbamate, which spontaneously decomposes to give a second molecule of ammonia and bicarbonate. The reaction products cause an overall pH increase that has negative effects both on human and animal health, as well as on the ecosphere [28,29]. The structures of urease from several bacteria and from higher plants have revealed the molecular architecture of the enzyme (Figure 1A) [28,29]. In the fully-conserved urease active site, two Ni(II) ions are bridged by the oxygen atoms of a carbamylated lysine residue and bound to two histidines. One Ni(II) ion is additionally bound to an aspartate carboxylate oxygen. The coordination geometry of the Ni(II) ions is completed by a water molecule bound to each metal ion and by a nickel-bridging hydroxide ion (Figure 1A) [28,29]. *In vivo*, holo-urease is post-translationally synthesized starting from apo-urease and following an activation process that involves CO_2_ uptake for lysine carbamylation, hydrolysis of GTP, and Ni(II) delivery into its active site. These events are typically carried out by four specific accessory proteins named UreD, UreF, UreG, and UreE [28]. The “classical” hypothesis for this process [28,30] involves the sequential binding of UreD, UreF, and UreG [or of a preformed aggregate of UreD, UreF, and UreG (UreDFG)] to obtain a pre-activation complex that carbamylates the active site lysine side chain and further binds Ni(II) ions delivered by UreE [28,30] through a route driven by GTP hydrolysis (Figure 1B, blue arrows path). Little is known on the functional properties of UreD [31], apparently the first protein that binds apo-urease. UreF is proposed to bind the urease-UreD complex through a direct interaction with UreD [32] and favors the formation of the urease-UreDFG complex [33]. UreG is responsible for coupling GTP hydrolysis to the process of urease activation and it is proposed to catalyze, in the presence of CO_2_, the formation of carboxyphosphate, an excellent carbamylation agent for the metal-binding lysine in the urease active site [33]. UreG is the first case of an intrinsically disordered enzyme [34], which can retain enzymatic activity owing to the rigidity of the active-site environment [35]. UreF has been also proposed to regulate the function and the folding of UreG, acting as a GTPase-activating protein (GAP) [36].

Finally, UreE is proposed to be the metallo-chaperone in charge of delivering Ni(II) ions [37], and actively transferring Ni(II) to urease apo-protein within the apourease-UreDFG complex in a GTP-dependent activation process [38]. Indeed, *in vivo* studies using yeast two-hybrid analysis [39,40] and coimmunoprecipitation assays [40], as well as calorimetry and NMR spectroscopy [41] indicated a direct interaction between UreE and UreG from *Helicobacter pylori* (*Hp*).

**Figure 2:**
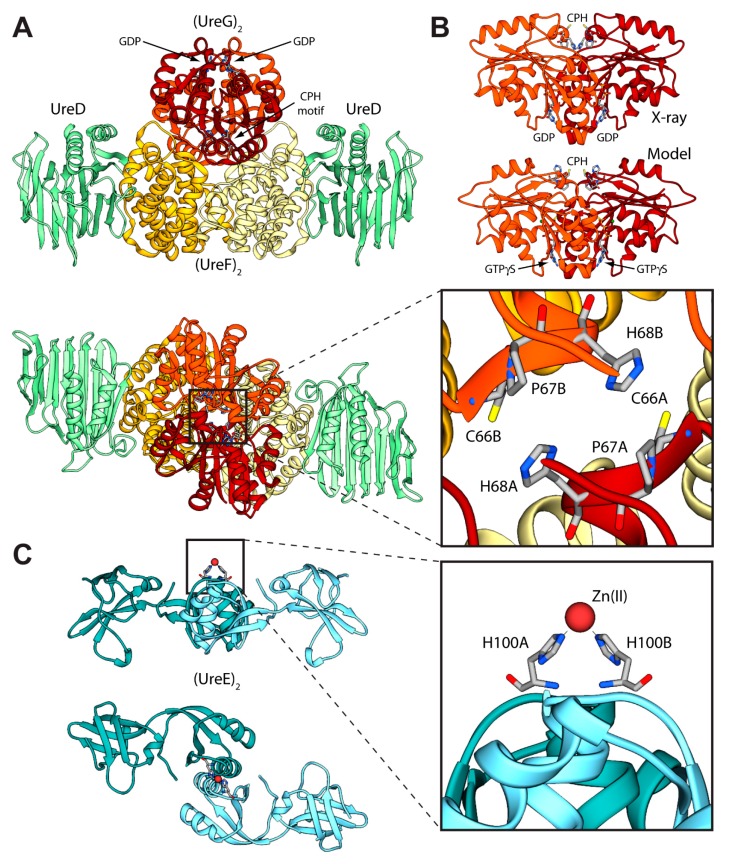
(**A**) Ribbon diagram of the (UreDFG)_2_ crystal structure from *H. pylori* (PDB id 4HI0) [44]. *Hp*UreD, *Hp*UreF, and *Hp*UreG chains are colored in light green, yellow and dark red, respectively, with the darker and lighter portions highlighting the different monomers. The views in the top and bottom panel are rotated by 90° along the horizontal axis. The inset reports the position of the residues forming the *Hp*UreG Cys-Pro-His conserved motif; (**B**) Comparison between the *Hp*UreG dimeric crystal structure, as found in the *Hp*(UreDFG)_2_ complex, and the homology model structure calculated in ref. [47]; (**C**) Ribbon scheme of the structure of *Sp*UreE homodimer (PDB id: 1EAR); the two monomers are colored in light blue, with the darker and lighter portions representing the two monomers. The views in the top and bottom panel are rotated by 90° along the horizontal axis. In the inset is reported the detail of the *Sp*UreE metal binding site.

Even though the structure of urease bound to any of the accessory proteins is not yet available, the crystal structure of the (UreF)_2_ homodimer [42] and the structure of the (UreDF)_2_ [43] and (UreDFG)_2_ [44] complexes from *H. pylori* have been recently reported (Figure 2A). The crystal structure of the *Hp*(UreDFG)_2_ complex contains two copies of each of UreF, UreD, and UreG, related by two-fold symmetry, forming a dimer of heterotrimers (Figure 2A). *Hp*UreF adopts an all-helical fold that consists of a bundle of nine α-helices arranged in an antiparallel fashion. The UreF dimerization occurs through α-helices 2, 3, 8 and 9 [42]. *Hp*UreD fold consists of 17 β-strands and two α-helices located near the *C*-terminus. The structure is dominated by two mixed strand β-sheets [43]. The topology of *Hp*UreG is characteristic of the SIMIBI class GTPases [45] and the protein was co-crystalized with one GDP molecule per monomer. The sequences of UreG feature a Cys-Pro-His (CPH) conserved motif that is fundamental for the metal binding properties of the protein [41,46]. In the *Hp*(UreDFG)_2_ structure (as well in previously calculated model structures [47,48]) the CPH motif is found in a cleft at the UreG dimerization interface (Figure 2A) [44]. Interestingly, despite the fact that *Sp*UreG and *Hp*UreG model structures previously published by our group were based on distant homologues [34,47,48,49], the experimental structure of *Hp*(UreG)_2_ found in the *Hp*(UreDFG)_2_ complex and the model structure of dimeric *Hp*UreG are very similar (Figure 2B), with a root mean square deviation (RMSD) of only 1.8 Å for the Cα atoms. Structural information on UreE proteins from various bacteria has been derived from numerous crystallographic studies: UreE from *Sporosarcina pasteurii* (formerly known as *Bacillus pasteurii*, *Sp*UreE) [50,51], *Klebsiella aerogenes* (*Ka*UreE) [52], and *H. pylori* (*Hp*UreE) [53,54] display a similar fold made by a symmetric homo-dimer, with each monomer composed of two domains connected by flexible linkers (Figure 2C). The fully conserved metal binding site is located on the surface of the *C*-terminal domain, at the dimerization interface. The *N*-terminal domain has been found in slightly different orientations with respect to the *C*-terminal domain, suggesting that some inter-domain conformational freedom is available for this protein, a feature possibly related to induced-fit processes that occur during the formation of protein-protein complexes involving the other urease chaperones [55]. On the other hand, this means that the UreE *N*-terminal domain conformations observed in the solid state could not be the same needed for the correct formation of the complex with UreG [41]. The entirety of the structural information from crystallography, together with UV-VIS spectroscopy, light scattering experiments, and GTPase activity assays performed on *Hp*UreG, recently suggested a new mechanism for the biosynthesis of the urease active site (Figure 1B, orange arrows path) [56]. In the new pathway, the Ni(II)-bound UreE dimer binds two apo-UreG monomers, facilitating GTP uptake by UreG in the presence of Mg(II) ions. The UreG binding to UreE can, in principle, occur either in a single or in a multistep process. In the (UreEG)_2_ complex, the Ni(II) ion is then translocated from UreE to UreG. Subsequently, the pre-formed (UreDF)_2_ complex competes with UreE for the Ni(II)-(UreG)_2_ dimer to form the supercomplex apo-urease/Ni(II)-(UreDFG)_2_. Finally, the GTP hydrolysis performed by UreG is catalyzed by KHCO_3_/NH_4_HCO_3_ to complete the nickel insertion into the apo-urease.

### 3.1. Calculation of the Structure of the UreEG Complex

**Figure 3:**
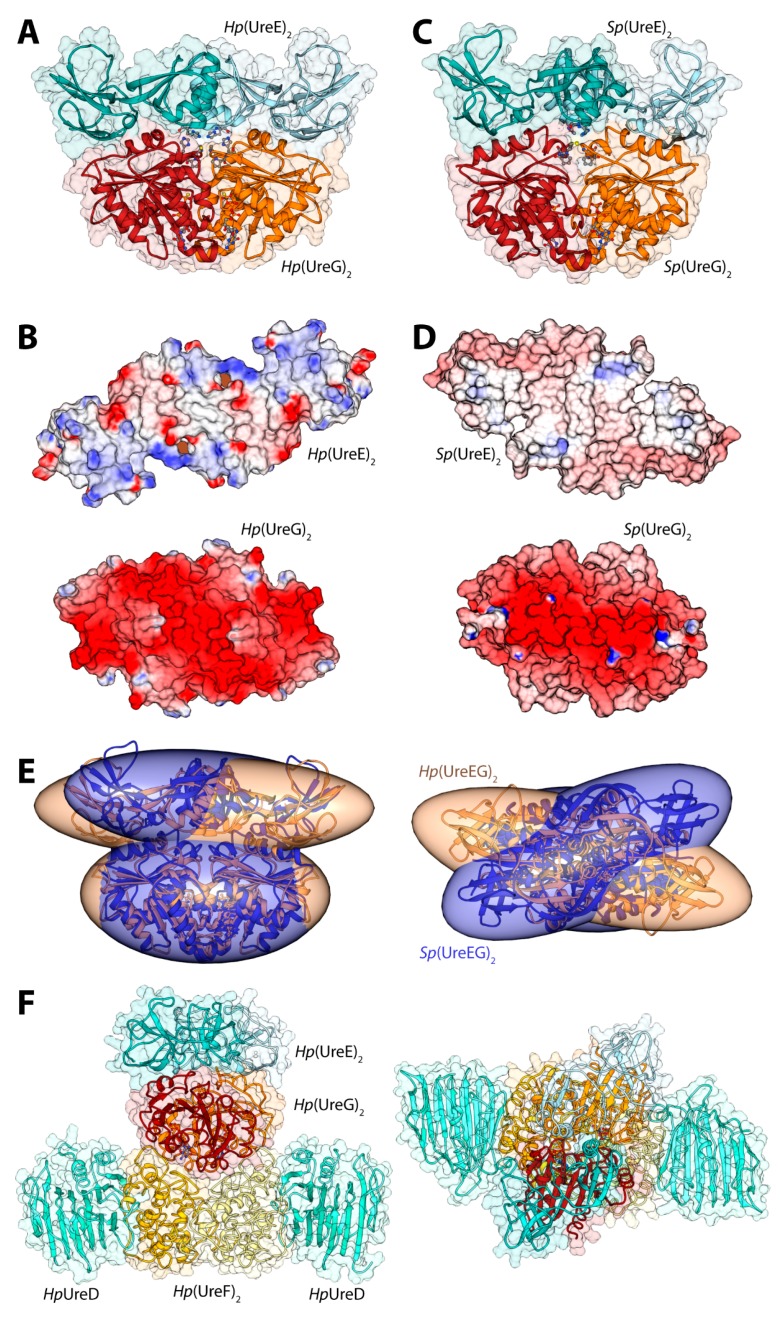
(**A**,**C**) Ribbon schemes and solvent-excluded surfaces of the model complexes of the *Hp*(UreEG)_2_ (**A**) and the *Bp*(UreEG)_2_ (**C**) structures. Ribbons are colored as in Figure 2. Residues found at the interface of the complex and known to be involved in metal binding (*Hp*UreE H102, *Sp*UreE H100 and UreG CPH motif residues), as well as the positions of the GTPγS and GDP molecules (bound to *Hp*UreG and to *Sp*UreG, respectively), are shown as ball and stick models and the atoms are colored accordingly to the atom type; (**B**,**D**) Solvent excluded surfaces of two components of the *Hp*(UreEG)_2_ (**B**) and the *Bp*(UreEG)_2_ (**D**) complexes orientated in order to expose the interaction surfaces. The surface is colored according to the surface electrostatic potential (blue, positive; red, negative); (**E**) Superposition of the structures and of the inertia ellipsoid of *Hp*(UreEG)_2_ (in orange) on the *Bp*(UreEG)_2_ (in blue) model complex. Left and right panels are rotated by 90° along the horizontal axis, keeping the UreG model as a reference; (**F**) Ribbon diagram and solvent excluded surface of the best *Hp*UreDFGE docked complex. Ribbons are colored as in Figure 2. Left and right panels are rotated by 90° along the horizontal axis.

The first attempt to model the UreE-UreG complex from *H. pylori* (*Hp*(UreEG)) was published in 2009 [41]. The calculation was prompted by a number of experimental observations: (i) *Sp*UreG and *Mycobacterium tuberculosis* UreG (*Mt*UreG) are dimers in native conditions, while *Ka*UreG and *Hp*UreG are monomeric proteins [47]; (ii) Zn(II)-driven protein dimerization occurs *in vitro* for *Hp*UreG, with one Zn(II) ion binding at the protein dimerization interface using the conserved cysteine and histidine residues from the CPH motif from each monomer [47]; (iii) Zn(II) and Ni(II) share the same binding site at the interface of the protein dimer in the *Sp*UreE crystal structure [50], with Zn(II) affinity ten times lower than Ni(II)-affinity for *Ka*UreE [57], while the thermodynamics of Zn(II) binding to *Hp*UreE is very similar to that of Ni(II) [41]; (iv) the *Hp*UreEG protein complex contains two monomers of *Hp*UreG dimerized onto one *Hp*UreE dimer [41], with an interaction stabilized by the presence of Zn(II) and not by Ni(II) [41], suggesting a role for Zn(II) in promoting the UreE interaction network. Thus, the hypothesis at the basis of the docking calculation was that the two conserved metal binding sites found on dimeric UreE and UreG are able to come in close contact during the formation of the *Hp*(UreEG)_2_ complex. The only UreE-available structures at that time were those from *S. pasteurii* and *K. aerogenes*, while there was no structure for UreG from any organism. Thus, the structures of *Hp*UreE and *Hp*UreG were modelled starting from homologue experimental structure [41]. In the case of *Hp*UreE the templates were the crystal structures of *Sp*UreE and *Ka*UreE (PDB id: 1EAR [50] and 1GMW [52], respectively) following a previously established procedure [55], while for *Hp*UreG the template structure was HypB from *Methanocaldococcus jannaschii* (*Mj*HypB, PDB id: 2HF8 [58]), an homologue GTPase involved in the biosynthesis of [Ni,Fe]-hydrogenase [47]. The RosettaDock software [59] was used to calculate an initial complex between the model structure of dimeric *Hp*UreG, and the central *C*-terminal domains of dimeric *Hp*UreE. This step allowed the calculations of a complex not biased by the conformation of the *Hp*UreE *N*-terminal domains. The protocol used by RosettaDock included (i) prepacking of the partners to remove clashes in the free monomers; (ii) global search of rigid-body orientations; and (iii) clustering of the low-energy models and selection of the largest cluster as prediction. The global search step starts from a large number of random initial orientations and brings the partners into glancing contact and removes clashes. It then proceeds by optimizing rigid-body orientation at a low-resolution level (where each amino acid side-chain is represented by a centroid pseudo-atom that is positioned according to an average position determined from a set of known structures from the PDB), and subsequently builds initial side-chain conformations using a Monte Carlo search through a backbone-dependent rotamer library. Finally, the protocol uses *ca.* 50 cycles of Monte Carlo minimization to optimize the side-chain and rigid-body degrees of freedom using a free energy function dominated by short-range Lennard-Jones and hydrogen-bonding interactions, and an implicit solvation model [59]. A search of 1000 complexes was carried out by randomly translating and rotating the initial positions of the interacting proteins. The complex with the best RosettaDock score was selected among all generated models for the subsequent refining cycle, carried out by applying 1000 times a perturbation to the starting structure. The Cα trace of this complex and the crystal structures of *Mj*HypB, *Sp*UreE, and *Ka*UreE were used as templates to build 200 structural models of the *Hp*(UreEG)_2_ complex using the Modeller software [60]. The best model was selected on the basis of the lowest value of the Modeller objective function. This modelling step allowed the addition of the *Hp*UreE *N*-terminal domains to the structure of the calculated model complex between the *C*-terminal domain of *Hp*UreE and *Hp*UreG. The final *Hp*(UreEG)_2_ model (Figure 3A) showed the two proteins facing each other along their extended axes, and only limited modifications of the proteins backbone, restricted both in extent and in topology distribution, were necessary in order to optimize the docking procedure. The central pocket formed on the *Hp*UreG surface around the conserved CPH motif matches the shape and volume of the protruding metal binding region on the surface of *Hp*UreE. The shallow crevice formed between the central *C*-terminal domain and the peripheral *N*-terminal domain of *Hp*UreE is filled with the bulge found on the surface of *Hp*UreG around the rim of the protein dimerization interface, and an overall continuous contact surface, with optimal electrostatic complementarity, was obtained (Figure 3B) [41].

In 2014, new data provided by NMR allowed a deeper characterization of the *Sp*UreE residues in contact with S*p*UreG [61]. These new findings, in general agreement with the proposed region of *Hp*UreE interacting with *Hp*UreG in the docking calculations performed earlier on the same proteins from *H. pylori* [41], prompted the calculation of the *Sp*UreEG complex driven by the NMR experimental information. The calculation used the latest published structure of *Sp*UreE (PDB id: 4L3K [51]) as well as a model of *Sp*UreG calculated based on its high degree of homology (sequence identity = 58%) with *Hp*UreG, whose crystal structure in complex with *Hp*UreF and *Hp*UreD had been released in 2013 [44]. In order to take into account the flexibility of the *Sp*UreE domains, the five low-frequency normal conformational modes of the dimeric *Sp*UreE crystal structure were calculated using the el Némo web-server [62] and a recently published protocol (see ref. [63] and below). The eleven protein conformations determined using this analysis, which comprised the starting structure as well as the ten structures derived by applying the perturbations consistent with each calculated normal mode to the starting structure, were utilized to build a library of structures to be used in the subsequent docking stages. The *Sp*UreE dimeric structure was docked onto the *Sp*UreG dimeric model complex using the data-driven docking program Haddock 2.1 [22,23] (see above for a general description of the program). For *Sp*UreE, the active residues were those identified by NMR chemical shift and signal intensity perturbations. For *Sp*UreG, the residues in the conserved CPH motif [34] and the most conserved residues on the same side of the protein determined using the server ConSurf [64,65,66] were used to guide the docking. The general features of the calculated *Sp*(UreEG)_2_ complex (Figure 3C) [61] are similar to those observed in the previously reported complex for *Hp*(UreEG)_2_ [41]. The *Sp*UreE residues in direct contact with *Sp*UreG are in good agreement with the experimental data, witnessing the correct outcome of the docking simulation [61]. The *Sp*UreE surface in the metal binding region is mainly hydrophobic, with some spots in correspondence of positively charged residues. The electrostatic potential mapped on the protein surface of *Sp*UreG shows that the interaction region is mainly negatively charged (Figure 3D). Therefore, the presence of a divalent cation bound to the *Sp*UreE metal binding site could efficiently change the electrostatic properties of the protein surface, allowing a more favorable interaction with the negatively charged surface of *Sp*UreG [61], consistently with the stabilization of the UreEG complex in *H. pylori* upon metal binding [41]. The main difference between the model complexes from *S. pasteurii* and *H. pylori* is an anticlockwise rotation of the *Sp*UreE dimer around the vertical axis of about 35 degrees (Figure 3E) [61].

### 3.2. Calculation of the Structure of the UreDFGE Complex

The calculation of the model of the *Hp*UreGE complex released in 2009 (see above) [41], together with the publication of the *Hp*UreDF crystal structure in 2011 (PDB id: 3SF5 [43]), provided the potential to investigate the structural propertied of the putative UreDFGE supercomplex using macromolecular docking, because all the pieces of the puzzle were finally available. A bioinformatics analysis conducted by combining the results obtained from ConSurf [64,65,66] and ProBis [67,68,69], together with experimental data from the mutagenesis studies conducted on UreG from *H. pylori* [43] and *K. aerogenes* [70], allowed us to identify a restricted set of residues not already involved in other protein-protein interactions on the surfaces of *Hp*UreF and *Hp*UreG. ConSurf calculates surface residue conservation, while ProBiS predicts surface regions containing structural similarity with known protein binding sites based on geometric and physicochemical parameters. Therefore, the *Hp*UreGE complex was docked onto the *Hp*UreDF complex using the docking program Haddock and a protocol identical to that utilized for the calculation of the *Sp*UreGE complex (see above) [61]. The structural models resulting from this docking process were clustered on the basis of their RMSD, allowing the identification of the two most populated clusters (composed of 58 and 51 models, respectively) at a similar Haddock score. The two clusters are indeed representative of the same *Hp*UreDF-*Hp*UreGE model complex because they differ only by the rotation of the symmetric *Hp(*UreG)_2_ homodimer by approximately 180 degrees along a vertical axis perpendicular to the surface of *Hp*UreF. In the resulting modeled structure of the *Hp*UreDFGE complex (Figure 3F), UreG is in direct contact with UreF through weak van der Waals interactions and a number of polar interactions. The shallow crevice formed at the interface between the two UreF monomers is in close contact with the convex surface of the UreG dimer (Figure 3F). Moreover, structural details in the UreF-UreG interaction surface supported the proposition of a role for UreF as a GTPase activating protein (GAP) [36]. Subsequent to the publication of the paper describing the model of the *Hp*UreDFGE complex [71], the crystal structure of the *Hp*UreDFG complex was released (PDB id: 4HI0, [44]). This structure showed that the general secondary and quaternary structure of *Hp*UreG agrees well with the model structure previously calculated [47], and that the binding site of *Hp*UreG onto the *Hp*UreDF complex surface is the same as the one identified earlier [71]. The crystal structure of the complex does not include *Hp*UreE. Unexpectedly, the main difference between the calculated model of the *Hp*UreDFGE complex and the crystal structure of the *Hp*UreDFG complex is the orientation of the *Hp*UreG protein dimer with respect to the *Hp*UreF interacting protein: *Hp*UreG interacts with *Hp*UreF using the surface patch that was predicted to interact with *Hp*UreE on the basis of experimental data [41]. In order to explain this difference, it has been proposed that *Hp*UreG, being an intrinsically partially-disordered protein, undergoes the known moonlighting behavior [72], changing its partner as needed along a metabolic pathway [56,71].

### 3.3. Calculation of the Structure of the Urease-UreDFG Complex

**Figure 4:**
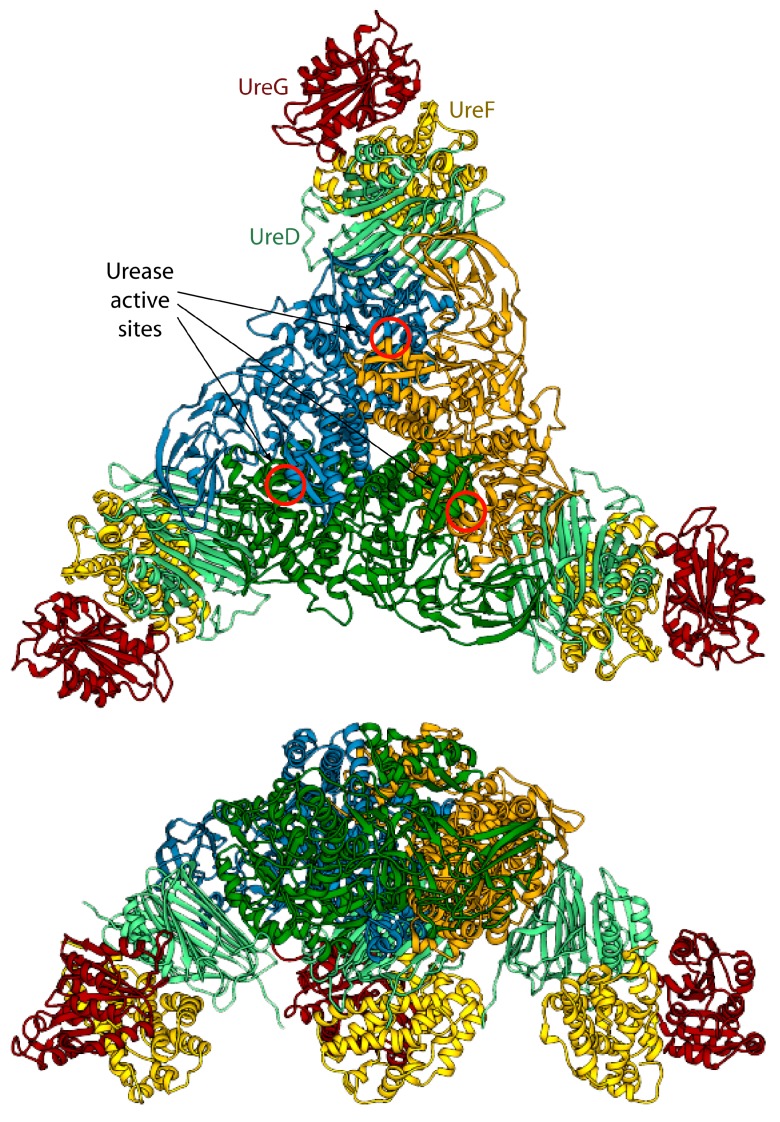
Ribbon scheme of the KAU–UreDFG model complex as calculated in ref. [73]. Ureases subunits and accessory protein are colored as in Figure 1 and Figure 2, respectively. The position of the KAU active sites is indicated by red circles. The views in the top and bottom panel are rotated by 90° along the horizontal axis.

In 2012, Ligabue-Braun *et al.*, attempted to build a model of the interaction between *K. aerogenes* urease (KAU) and three of the required accessory proteins *Ka*UreD, *Ka*UreF, and *Ka*UreG, in their monomeric oligomerization state [73]. KAU (as well as *S. pasteurii* urease) is a trimer of trimers formed by the three structural urease proteins: UreA, UreB, and UreC [28,29]. The adopted strategy included (i) the homology modelling of the structure of *Ka*UreG based on the structure of *Mj*HypB (the structure of *Hp*UreG was not available at the time of the publication) and the homology modelling of the monomeric *Ka*UreD and *Ka*UreF based on the structure of *Hp*(UreDF)_2_ structure (PDB id: 3SF5, [43]); and (ii) a stepwise docking procedure in which the monomers of *Ka*UreD, *Ka*UreF, and finally *Ka*UreG were docked on the crystal structure of KAU [73]. All the docking steps, with the exception of the last stage (binding of *Ka*UreG), were performed without imposing any restraints. The authors used three different rigid-body docking programs: PatchDock [74], Hex [75], and PIPER [76] via ClusPro 2.0 [77]. PatchDock divides the surface of the two interacting biomolecules into patches according to the surface shape. The obtained patches correspond to patterns that visually distinguish between puzzle pieces. Once the patches are identified, they can be superimposed using shape-matching algorithms that go through three major stages: (i) molecular shape representation; (ii) surface patch matching; and (iii) filtering and scoring. Both Hex and PIPER use a FFT method [78] to perform a rigid-body search of the proteins’ orientations. Hex accelerates the search by using an expansion of the molecular surface and electric field in spherical harmonics. Fourier correlations between the expansion coefficients are used to simplify the problem of calculating the complementarity between the surfaces in different orientations to that of look up in a table of pre-calculated overlap integrals [79]. PIPER approximates the interaction matrix by its eigenvectors corresponding to the few dominant eigenvalues, resulting in an energy expression written as the sum of a few correlation functions, and by using repeated FFT calculations [76]. In all cases, the results were subsequently clustered with MMTSB Tool Set [80] by hierarchical clustering based on mutual RMSD and evaluated in terms of relative energy with FoldX [81]. The obtained complexes (Figure 4) were checked against experimental small-angle X-ray scattering (SAXS) profiles obtained for the *K. aerogenes* KAU-UreDFG supercomplex [73] as well as against results derived from cross-linking, mutagenesis, and pull-down experiments. Theoretical SAXS profiles were calculated using the FoXS server [82] and compared to the experimental data for the KAU-*Ka*UreD and KAU-*Ka*UreDF complexes [83].

The model of the supercomplex is in agreement with pull-down [46] and cross-linking [32] assays. On the other hand, the hinge region of *Ka*UreB (residues 1–19), which is essential for proper urease activation [84], does not bind directly to any accessory protein. The authors suggested that this region could be required for a proper “gating” of the active site for activation [73]. Furthermore, the accessory proteins bind far away from the urease active site, and a clear pathway for nickel traffic towards the active site was not identified [73]. Finally, the spatial assembly of the urease-UreDFG supercomplex observed in the case of *K. aerogenes* could not be a general case. Indeed, ureases from *H. pylori* or jack bean assume different quaternary structures (the former is a tetramer of trimers of dimers, while the latter is a dimer of trimers of monomers, see Figure 1A) and in such arrangements there is no room for the accessory proteins in the same position of the complex proposed by Ligabue-Braun *et al.* [73]. The authors suggest that *H. pylori* represents an exception among bacterial ureases, with such difference reflected in a different structural organization of its activation complex, while for plant ureases they hypothesize that the trimer of monomers is activated before the formation of the final protein structure.

## 4. *Hp*Fur-DNA and *Hp*NikR-DNA Complexes

Fur and NikR are two important metal-responsive regulators involved in iron and nickel homeostasis and function by controlling gene expression in *H. pylori*. Fur and NikR can bind independently at distinct operators, and also compete for overlapping operators in some co-regulated gene promoters, suggesting a link between iron and nickel regulation [85].

### 4.1. HpFur-DNA Complexes

Iron holds a central position in the host-pathogen interplay in mammals and plants [86]. For instance, iron deficiency is the most common nutritional stress in aquatic environments [87]. In order to manage iron limitation, cells have developed a large number of responses that enable maintenance of iron homeostasis through precise changes in gene expression. The latter allow the cells to adapt their physiology to the environment and to prevent nutrient overloads that would be highly poisonous or lead to oxidative stress [88]. The control of iron metabolism and its coupling with regulation of defenses against oxidative stress is carried out by Fur in most prokaryotic organisms. In *H. pylori*, Fur is able to act as a transcriptional commutator switch that exploits the alternative readout of DNA grooves to mediate opposite output regulation for the same input signal [63]. In particular, when Fe(II) ions are abundant, gene transcription is repressed by *Hp*Fur in an iron-dependent manner, conforming to the classic Fe(II)-Fur (holo-Fur) repression paradigm [89], in which the iron ion acts as corepressor. Accordingly, the iron-repressible Fur targets include genes involved in Fe(II) ions uptake, such as the prototypical *frpB1* gene, which needs to be expressed only under iron-starving conditions [90]. On the other hand, when iron is scarce, apo-*Hp*Fur represses transcription of a different set of genes, including the iron-inducible *pfr* gene, which codes for a ferritin involved in iron storage, thus demanding derepression only under iron-replete conditions [91]. In this case, the Fe(II) cofactor ion acts as an inducer, rather than a corepressor.

**Figure 5:**
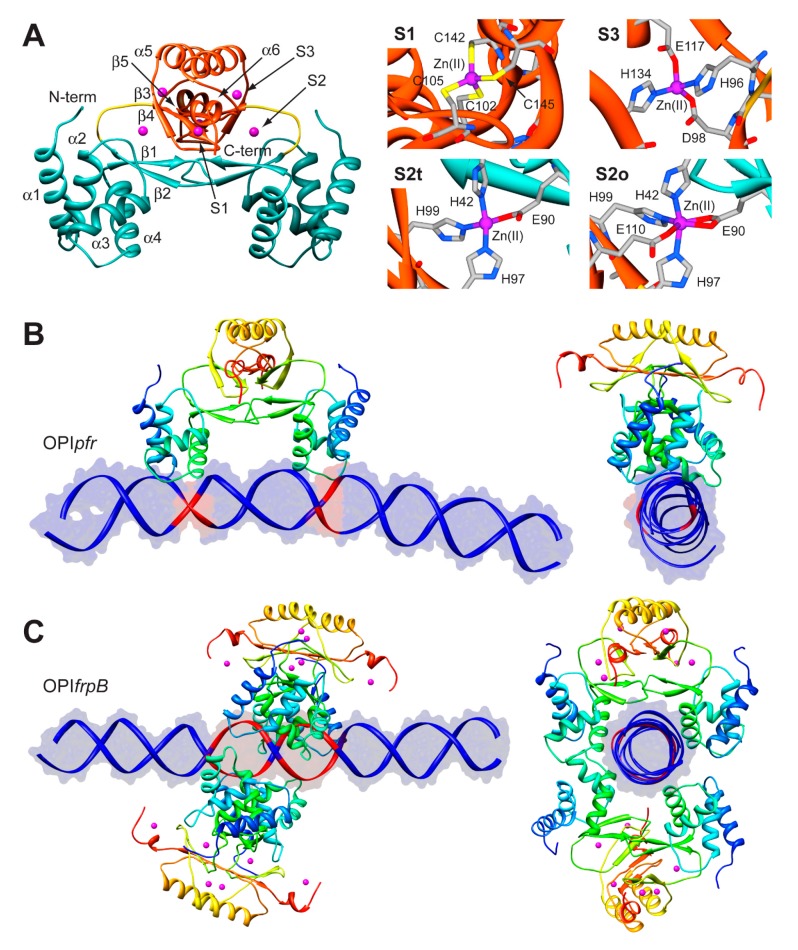
(**A**) Ribbon scheme and details of the metal binding sites of *Hp*Fur crystal structure. Ribbons for DBD and MBD in left panel are colored in cyan and in orange, respectively, while the unstructured region linking the two domains is in yellow. Zn(II) ions are in purple. In the right panels, metal binding residues are reported as sticks colored according to atom type; (**B**,**C**) Model structures of the *Hp*Fur-DNA: best docking models resulting for *Hp*Fur-OPI*_pfr_* (**B**) and Fur-OPI*_frpB_* (**C**) complexes. The protein is reported as ribbon diagram colored from blue in the proximity of the *N*-terminal to red at the *C*-terminus. Zn(II) ions are in purple. The DNA is in blue, with the exception of the active residues that are in red.

The structure of Fur is a homo-dimer built through a head-to-head interaction of the C-termini of the protein. Each monomer is formed by a DNA-binding domain (DBD) at the *N*-terminal and a metal binding domain (MBD, also called dimerization domain) at the *C*-terminal (Figure 5A). Each Fur monomer binds metal ions in three sites: a regulatory site that can bind one Fe(II) (site S1), a structural site binding one Zn(II) (S2), and a not fully conserved site with uncertain function (S3) (Figure 5A) [92]. Size exclusion chromatography indicated that *Hp*Fur is a dimer in solution and that it is able to tetramerize, and further multimerize, in the presence of divalent metal ions such as Fe(II) and Mn(II), even in the absence of DNA [63].

To gain insight in the molecular mechanism responsible for the distinctive recognition of apo- and holo-operators, docking simulations were run using the program Haddock and the crystal structure of holo-*Hp*Fur (C78S and C150S mutant, PDB code: 2XIG [93]). In this structure there are three Zn(II) ions bound to each of the metal binding site. Curiously, in the regulatory site the Zn(II) ion is found in two different coordination geometries: in one chain of the dimer the metal ion is tetra-coordinated (S2t in Figure 5A), while in the second chain the Zn(II) ion adopts a distorted octahedral geometry (S2o). This discrepancy in the coordination geometry can be due to the presence of Zn(II) ions instead of the physiological Fe(II) ions in the crystallization milieu. Thus, the first step was to use homology modelling to reconstruct a model of the wild type holo-*Hp*Fur featuring both the regulatory metal binding sites in a tetrahedral coordination geometry [63]. The calculation was performed using the Modeller software [60] and the coordination geometry was induced by introducing appropriate symmetry, bond and angle constraints. The model structure of apo-Fur was obtained by depletion of Zn(II) ions from the model structure of holo-Fur. In order to take into account the experimentally-observed mobility of the DBDs with respect to the MBD [92,93], two libraries of possible Fur conformations were created by analyzing the low-frequency normal modes of the apo- and holo- protein models by using the elNémo web server [62]. In particular, eleven protein conformations (the starting structure, in addition to the structures derived by applying to the starting structure, the perturbation of every calculated normal mode in the two possible directions) determined using this analysis were utilized to build a library of structures for each *Hp*Fur metalation state to be used in subsequent docking calculations together with the apo- and holo-operator (OPI*_pfr_* and OPI*_frpB_* hereafter, respectively) [63]. A starting model for the unbound DNA operators were generated using the DNA analysis and rebuilding software 3DNA implemented in the 3D-DART server [27]. The docking calculations were carried out by considering all the possible combinations between apo- and holo-*Hp*Fur and the two operators (*i.e.*, apo-*Hp*Fur/OPI*_pfr_*, holo-*Hp*Fur/OPI*_pfr_*, apo-*Hp*Fur/OPI*_frpB_*, holo-*Hp*Fur/OPI*_frpB_*). In Fur, α-helix 4 is known to be part of the DNA recognition process [94] and in particular the conserved Y65 (Y55 in *E. coli* numeration) [95] appears to be involved in the binding. Thus, the calculations on the protein side were guided defining as “active” the residue Y65, while the surface residues around it were considered “passive” residues [63]. In the apo- and holo-operators, the base pairs identified in footprinting assay were defined as “active” (bases −19, −18, −7, −6 for OPI*_pfr_* and from −25 to −21 and from −19 to −15 for OPI*_frpB_*, see Figure 5B,C) [63]. In all cases, the docking protocol was optimized by running calculations in the presence and absence of additional restraints on the symmetry of the complex and on the DNA bases [63].

In the case of OPI*_pfr_* complexes, the best result was obtained for the apo-Fur/OPI*_pfr_* model (Figure 5B) with the application of restraints to take into account the symmetry of the complex and the DNA bases known to be involved in the interaction. The calculated protein-DNA complex features the axis connecting the two DNA-binding domains nearly parallel to the DNA major axes. The Fur DNA-binding domains insert the loop between α-helices 1 and 2, as well as the first five residues of α-helix 4, in the major groove of the apo-operator in correspondence with the two regions identified by footprinting assay. The apo-operator assumes a convex conformation with respect to the Fur position, broadening the major grooves exposed to the protein and thus promoting protein binding. On the other hand, in simulations carried out on the OPI*_frpB_* operator, we observed a different orientation of the protein with respect to the considered DNA fragment. In all the docking calculations involving OPI*_frpB_*, *Hp*Fur clamps the holo-operator positioning the axis connecting the DNA-binding domains perpendicularly to the DNA major axis. These results, together with the experimental observation of the formation of a complex involving OPI*_frpB_* and tetrameric holo-*Hp*Fur, prompted us to perform a simulation in which we included two *Hp*Fur dimers together with the holo-operator. In these simulations we included additional restraints derived from the electrostatic properties of the residues found on the surface of the DNA-binding domain. In particular, our hypothesis was based on the observation that K24 and N58 are placed in good position for inter-protein interaction in the case of the formation of a tetramer build by connecting two Fur dimers across the DNA-binding domain. In the resulting best complex (Figure 5C), the two Fur dimers envelop the DNA using both the DNA-binding domain and the dimerization domain, completely covering the large region of the operator identified by footprinting assay. The Fur tetramer does not bind DNA perpendicularly to the operator major axes, but is tilted by about 30 degrees, allowing both Fur dimers to putatively interact with DNA regions that are feebly detected in the footprinting assay (Figure 5C) [63].

### 4.2. HpNikR-DNA Complex

**Figure 6:**
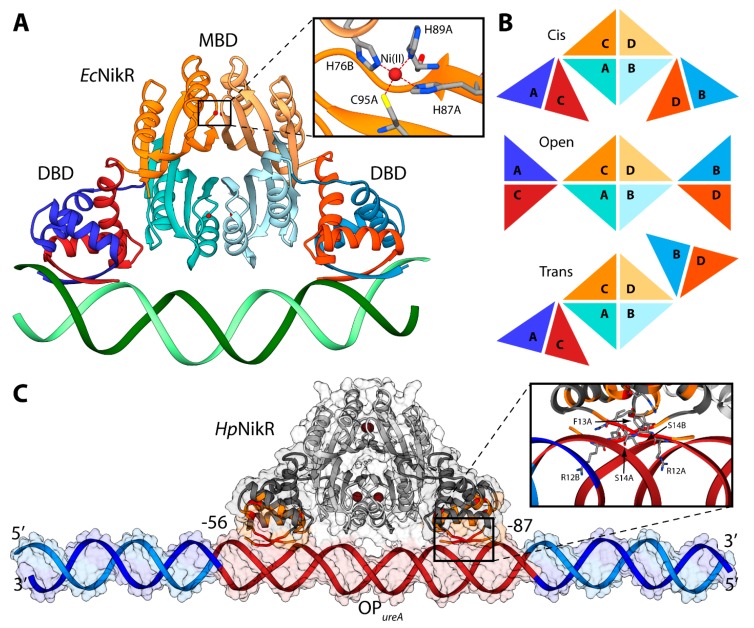
(**A**) Ribbon scheme of the crystal structure of holo-*Ec*NikR in complex with DNA. DBD are in orange and blue, while MBD are in light orange and light orange. Nickel ions are reported as dark red spheres and DNA is in green. The inset reports the details of the Ni(II) binding site. Nickel binding residues are reported as sticks colored according to atom type; (**B**) Schematic representation of the conformations of NikR found in the available crystal structures; (**C**) Ribbon diagram and solvent-excluded surface of the model structure of the *Hp*NikR-DNA complex. *Hp*NikR ribbons are colored in dark and light grey in the DBD and MBD, respectively. Active and passive residues used to guide the docking are in red and orange, respectively. Ni(II) ions are reported as dark brown spheres. The DNA ribbons are in dark red for the *Hp*NikR operator and in light and dark blue for the terminal regions. The inset shows the details of *Hp*NikR residues in the DBD in direct contact with the DNA major groove.

NikR is a transcription factor that regulates the expression of genes coding for proteins involved in nickel metabolism [28,96]. It is a highly homologous protein, found in *ca.* 30 species of bacteria and archea. The Ni(II)-bound NikR from *E. coli* (*Ec*NikR) binds to DNA and represses the transcription of the nikABCDE operon, which codes for a specific Ni(II) membrane uptake ABC transporter [97,98]. On the other hand, NikR from *H. pylori* (*Hp*NikR) is a pleiotropic regulator of several genes, acting as a nickel-dependent repressor of *Hp*NikR itself and of the Ni(II) permease NixA, as well as a nickel-dependent activator of urease operon [99,100]. Numerous crystal structures of NikR [101,102,103] have established that this protein is a homo-tetramer, made of a dimer of dimers, constituted by two domains (Figure 6A). One domain is the central metal binding domain (MBD), made of the *C*-terminal portion of the protein responsible for tetramerization. This domain hosts four regulatory metal binding sites symmetrically located at the tetramerization interface, where Ni(II) ions bind three fully conserved histidines and one cysteine residues in a square planar coordination geometry (HHHC site hereafter, Figure 6A). Some crystal structures of full-length *Hp*NikR [101,103], showing partial occupancy of the square planar HHHC sites as well as additional Ni(II) binding sites, have been interpreted as indicating means for *Hp*NikR to recognize different DNA sequences depending on the level of Ni(II) concentration and cytoplasmic pH, but no evidence has been obtained so far to support this hypothesis The MBD is flanked by two peripheral DNA binding domains (DBD), separated by flexible linkers. Each DBD is made of the dimerization of the *N*-terminal portion of the protein and features a ribbon-helix-helix motif for DNA binding, typical of prokaryotic transcription factors [104], in which two anti-parallel *N*-terminal β-strands from opposite protomers make a two-stranded anti-parallel β-sheet that contacts the major groove (Figure 6A). Three distinct conformations of NikR have been observed in the solid-state, characterized by the position of the DBDs with respect to the central MBD, depending on the conformation of the flexible link between the domains: *cis*, *open*, and *trans* (Figure 6B). The crystal structure of the *Ec*NikR-DNA complex has shown that the *cis* conformation of the Ni(II)-bound protein is able to bind DNA with the MBD that keeps DBDs at the right distance to contact one-half-site of a two-fold symmetric DNA operator (Figure 6A) [105]. The Ni(II) binding to the MBD of *Hp*NikR results in an increase of the protein affinity to DNA, as determined by calorimetric titrations [106]. The way through which Ni(II) binding propagates the information for DNA binding at the MBD along the protein chain to the DBDs, has been investigated both experimentally [100,107,108], and computationally, using atomistic molecular dynamics simulations [100,109,110]. The conclusions suggest the occurrence of an ensemble of interconverting structures in solution, spanning the *open*, *trans* and *cis* conformers, both for the apo and the holo-protein. This implies that Ni(II) binding does not induce a conformational rearrangement of the protein towards a specific *cis* conformation able to bind DNA, but rather unlocks the movement of the two peripheral *N*-terminal DNA-binding domains with respect to the central *C*-terminal metal binding domain [100,110].

Some molecular details of the *Hp*NikR interaction with the urease operator OP*_ureA_*, based primarily on NMR spectroscopy in solution, have recently been reported [111]. Considering that the size of the full-length protein prevents the characterization of the *Hp*NikR-OP*_ureA_* interaction using only NMR, the two protein domains were investigated separately. An NMR-based analysis of the perturbations, induced on the DBD by the presence of OP*_ureA_*, provided information useful to guide the subsequent docking calculation aimed to build a computational model of the *Hp*NikR-OP*_ureA_* complex [111]. The homology model of the holo-*Hp*NikR in the *cis* conformation was calculated in a previous study [100]. The modeling was based on the structure of NikR from *E. coli* in the cis conformation (PDB ID: 2HZV, resolution 3.00 Å) [105]. The alignment between the sequences of NikR from all the available crystal structures (*H. pylori*, *E. coli*, and *P. horikoshii*) was produced using ClustalW [112] and manually adjusted in order to match the primary and secondary structure of the proteins. The obtained alignment was used to calculate 50 structural models of the *cis* conformation of tetrameric *Hp*NikR using the program Modeller [60]. Four square-planar Ni(II) ions were included in the modeling, bound in the well-known metal binding sites involving H88 from one monomer and H99, H101 and C107 from the adjacent monomer [100,111]. A starting model for the unbound OP*_ureA_* operator was generated using the DNA analysis and rebuilding software 3DNA implemented in the 3D-DART server [27]. OP*_ureA_* comprises nucleotides from −56 to −87 with respect to the urease operon transcriptional start site in *H. pylori* strain G27 [111]. In order to avoid biasing effects due to the highly charged DNA termini, twenty nucleotides were added to each side of the operator by using the *H. pylori* strain G27 genome. The model was generated in the canonical B-DNA conformation. The holo-*Hp*NikR model structure in the *cis* conformation was docked onto OP*_ureA_* using the data-driven docking program HADDOCK 2.1 and the same protocol used previously in the case of Fur (see ref. [63] and above). The calculation was guided by selecting the protein residues found by NMR to be involved in the interaction with DNA (R12, F13, S14, V15, S16, S36, R37, L40), as well as the operator’s nucleotides (from −56 to −87 with respect to the urease operon transcriptional start site in *H. pylori* strain G27).

The NMR-based docking model of the interaction complex between the full-length *Hp*NikR and OP*_ureA_* (Figure 6C), obtained using the NMR-based constraints described above, has strong analogies with the crystal structure of the analogous complex experimentally determined in *E. coli* [105]. The major axes of *Hp*NikR and OP*_ureA_* are almost parallel, with the latter uninterruptedly making contacts with *Hp*NikR, in agreement with DNAase I footprinting assays [106]. The DNA major axis is slightly bent, even though not as much as in the *E. coli* structure, while the major groove is more open in the region of interaction with the DBD domain, causing in turn shrinkage of the minor groove in the same region (Figure 6C). *Hp*NikR appears to interact with DNA mainly by inserting residues 12–14 of the DBD into the major groove in the proximity of nucleotides positioned from −24 to −26 and from −80 to −82 with respect to the urease operon transcriptional start site in *H. pylori* strain G27 (Figure 6C) [111].

## 5. Perspectives

The determination of the structure of the complexes between proteins, as well as between proteins and gene transcription operators, is an important step not only in the advancement of the basic knowledge of a metabolic process, but also in the identification of new targets for the development of new drugs. Moreover, several diseases are caused by alterations of transcription and/or protein-DNA interactions, making these mechanisms highly attractive targets for therapy [113].

Current methods and libraries for drug discovery work well with a limited number of targets, such as enzymes, ion channels or receptors, which feature a well-defined and structurally-stable concave binding sites. The G protein-coupled receptors (GPCRs), protein kinases and proteases super-families are good examples of this kind of targets with “druggable” characteristics [114,115,116,117,118]. Targeting protein-protein interfaces (PPIs) of multi-protein complexes that mediate cell regulation (long regarded by many as “undruggable”) has become a subject of intense activity in recent years [119]. Within protein-protein complexes of known structure, PPIs are generally large (*ca.* 1500–3000 Å^2^), flat, and relatively featureless [120], making the design of small molecule antagonists a challenging task. Furthermore, in the case of protein-DNA interactions, many factors have led to consider transcription as“undruggable” [121]. Indeed, several DNA binding proteins lack ligand-binding domains or intrinsic enzymatic activities [121]. Moreover, some of the protein domains involved in DNA recognition or protein recognition are intrinsically disordered in the absence of interacting partners [122,123]. Finally, the complexity of the interactions involving multi-point contacts over large surfaces, the lack of defined pockets suitable for a rational design of small molecules, and the presence of metal ions are critical issues that need to be addressed [121].

The development of protocols to mimic these difficult interactions is a key answer to these pressing needs, with the potential to offer the molecular details of new druggable targets. First it has been recognized that energetic hotspots contribute much of interface interaction free energy in many PPIs [124], but also it has been noted that many interactions involve continuous epitopes constituting defined grooves or series of small specific pockets [125]. Such observations lead to the development of stapled α-helical peptides and other proteo-mimetic approaches to drugging interfaces [126]. In a different approach, it has been noted that fragments might gain low-affinity “footholds” on PPIs, and that these might be elaborated to apt modulators of multi-protein assemblies where knowledge of the structures of the complexes is available [114]. In the latter examples, the experimental determination or the computational prediction of the structural details of the protein-protein or the protein-DNA interface is mandatory to the study of the interaction interfaces. In this aim, the macromolecular docking protocols discussed in this review have evolved in the last twenty years thanks to the development of new algorithms and the availability of more computational power, and have become an important complement to the experimental techniques.
